# Toxicity report of once weekly radiation therapy for low-risk prostate adenocarcinoma: preliminary results of a phase I/II trial

**DOI:** 10.1186/1748-717X-6-112

**Published:** 2011-09-09

**Authors:** Cathy Menkarios, Éric Vigneault, Nicolas Brochet, David HA Nguyen, Jean-Paul Bahary, Marjory Jolicoeur, Marie-Claude Beauchemin, Hugo Villeneuve, Thu Van Nguyen, Bernard Fortin, Carole Lambert

**Affiliations:** 1 Department of Radiation Oncology, Hôpital Maisonneuve-Rosemont, Montréal, Québec, Canada; 2 Department of Radiation Oncology, Centre hospitalier universitaire de Québec, Québec, Québec, Canada; 3 Department of Radiation Oncology, Complexe hospitalier de la Sagamie, Chicoutimi, Québec, Canada; 4 Department of Radiation Oncology, Centre hospitalier de l'Université de Montréal, Montréal, Québec, Canada

**Keywords:** prostate cancer, radiotherapy, hypofractionation, toxicity

## Abstract

**Background:**

Increasing clinical data supports a low α/β ratio for prostate adenocarcinoma, potentially lower than that of surrounding normal tissues. A hypofractionated, weekly radiation therapy (RT) schedule should result in improved tumour control, reduced acute toxicity, and similar or decreased late effects. We report the toxicity profile of such treatment.

**Materials and Methods:**

We conducted a multi-institution phase I/II trial of three-dimensional conformal radiation therapy (3D-CRT) for favourable-risk prostate cancer (T1a-T2a, Gleason ≤ 6 and PSA < 10 ng/ml). RT consisted of 45 Gy in nine 5 Gy fractions, once weekly. Primary end-points were feasibility and late gastrointestinal (GI) toxicity (RTOG scale), while secondary end-points included acute GI toxicity, acute and late genitourinary (GU) toxicity, biochemical control, and survival.

**Results:**

Between 2006 and 2008, 80 patients were treated. No treatment interruptions occurred. The median follow-up is 33 months (range: 20-51). Maximal grade 1, 2, and 3 acute (< 3 months) GU toxicity was 29%, 31% and 5% respectively (no grade 4). Acute GI grade 1 toxicity was reported in 30% while grade 2 occurred in 14% (no grade 3 or 4). Crude late grade ≥ 3 toxicity rates at 31 months were 2% for both GU and GI toxicity. Cumulative late grade ≥ 3 GI toxicity at 3 years was 11%. Two patients had PSA failure according to the Phoenix definition. The three-year actuarial biochemical control rate is 97%.

**Conclusions:**

Weekly RT with 45 Gy in 9 fractions is feasible and results in comparable toxicity. Long term tumour control and survival remain to be assessed.

## Background

In recent years, there has been increasing interest in hypofractionated radiation therapy (RT) for prostate cancer. Using the linear-quadratic (LQ) model for the effect of RT on tumour, emerging data supports a low alpha/beta (α/β) ratio for prostatic adenocarcinoma cells. Values of α/β ranging from 1.2 to 4 have been reported [1-8], with most data supporting values at the lower end of this spectrum. Furthermore, the α/β ratio for late rectal effects may be higher (around 4-6) [5] than the value of 3 for other late tissue effects. In this case, the LQ model predicts that a hypofractionated regimen would result in superior tumour control with a similar rate of late toxicity *or *lower late toxicity with a similar tumour control rate. Thus, a favourable therapeutic ratio can potentially be achieved by delivering a small number of larger fractions. However, hypofractionation can lead to more severe acute effects and increased consequential late damage. This can be prevented by increasing the time between fractions, which allows for normal tissue recovery without compromising efficacy when the tumour α/β is low.

In addition to the possible radiobiological benefits, hypofractionated RT with fewer fractions allows for increased patient convenience and minimal disruption to their lives. Other potential benefits are reduction in treatment costs for centralized health care systems and shortening of waiting lists in high volume treatment centers.

In 2006, we opened a phase I-II prospective trial of hypofractionated 3D-CRT for favourable-risk prostate cancer patients. The regimen consisted of 45 Gy in 5 Gy fractions, given once a week over nine weeks (57 days). Using the LQ model without time correction, this corresponds to a biologically equivalent dose (BED) of 83.6 Gy in 2 Gy fractions (EQD2) assuming an α/β ratio of 1.5. The BED for late effects on normal tissue is 72 Gy in 2 Gy fractions assuming an α/β ratio of 3.

Our findings of acute toxicity and preliminary late toxicity results are reported here, along with a comparison with other clinical hypofractionated studies in prostate cancer.

## Methods

### Study Population

Eligible men had histologically confirmed prostate adenocarcinoma with favourable-risk features defined as: clinical stage T1-T2a according to the American Joint Committee on Cancer (AJCC) [9], pre-treatment PSA ≤ 10 ng/ml, and Gleason Score (GS) ≤ 6. Exclusion criteria were: active inflammatory bowel disease, prior malignancy (other than non-melanoma skin cancer) treated less than 5 years prior to study enrolment, prior pelvic RT, previous or concurrent hormone therapy, previous therapy for prostate carcinoma, serious medical or psychiatric illness precluding compliance to protocol, and patients for whom prophylactic treatment of seminal vesicles was deemed necessary by the radiation oncologist.

The protocol was approved by the clinical research and ethical committees of participating institutions. Written informed consent was obtained from all patients before study entry.

Staging chest x-ray, bone scan and pelvic computed tomography (CT) scan were not compulsory, and were left at the discretion of the treating physician.

### Planning and Treatment Regimen

All patients were simulated in the supine position with a personalized immobilization device (Vac-Lok cradle). They were instructed to have a full bladder for the planning CT and before each treatment. No bowel preparation was used. An urethrogram was used for planning CT, and a slice thickness of ≤ 5 mm was obtained through the region that contained the target volumes and organs at risk (OAR).

The clinical tumour volume (CTV) was defined as the entire prostate. It extended inferiorly to 9-10 mm above the tip of the urethrogram. The planning target volume (PTV) was obtained by expanding the CTV radially with a 1.0-1.5 cm margin on all sides, except posteriorly where the margin was 0.5-1 cm. While these margins are slightly larger than those used in other protocols, they were deemed necessary to account for possible increased intrafraction motion due to longer treatment time (5 Gy/fraction). Moreover, only 9 fractions were given per patient and the investigators were intent on minimizing the risk of geographical miss. After 35 Gy (7 fractions), the margin for the PTV could be reduced to 0.5-1 cm in all directions, for example if the patient had been shown to require only small daily shifts up to then, and the investigator felt it safe to use current daily-imaging margins of 0.5-1 cm.

Elective seminal vesicles or pelvic irradiation was not permitted. The following OAR were outlined as solid structures on the planning CT: rectum from the anal sphincter to the rectosigmoid flexure, bladder, and femoral heads.

Patients received 45 Gy in nine weekly fractions of five Gy each over 9 consecutive weeks (total of 57 days) using 3D-CRT. The dose was prescribed at the isocenter, such that 100% of the PTV received ≥ 95% of the prescribed dose and that no region in the field received greater than 107% of the prescribed dose, as per ICRU recommendations. An isocentric technique of 5, 6, 7 or 9 fields was used. Intensity-modulated radiation therapy (IMRT) was not permitted. All patients were treated with a ≥ 10 MV linear accelerator. A daily localization procedure was mandatory using either implanted fiducial gold markers or transabdominal ultrasound (B-mode Acquisition and Targeting).

Dose constraints to OAR were based on the RTOG P0126 protocol, and estimated using the linear-quadratic model assuming an α/β ratio of 3 for late effects on normal tissue (Table 1).

**Table 1:** OAR dose constraints (assuming α/β ratio of 3 for rectum and bladder)

Organ	Threshold dose (Gy)	Volume above limit (%)
Bladder	49 45 40	15 30 50

Rectum	46 43 37	15 30 50

Right/left femoral heads	32.5	0

### Study Endpoints

Primary end-points were maximal late rectal toxicity (occurring more that 6 months after treatment) assessed using the RTOG scale [10] (Table 2), and feasibility, defined as the proportion of enrolled patients who completed treatment. Secondary end-points included acute rectal and urinary toxicity, late urinary toxicity, biochemical disease free-survival (bDFS), DFS and overall survival (OS). Biochemical failure was defined as per the Phoenix criteria as the postradiotherapy nadir plus 2 ng/ml.

**Table 2:** Appendix 1 - RTOG late toxicity scale

RTOG GRADE					
	**0**	**I**	**II**	**III**	**IV**

Bladder	None	Slight epithelial atrophy; minor telangiectasia (microscopic hematuria)	Moderate frequency; generalized telangiectasia; intermittent macroscopic hematuria	Severe frequency & dysuria; severe telangiectasia; frequent hematuria; reduction in bladder capacity (< 150 cc)	Necrosis/Contracted bladder (capacity < 100 cc); severe hemorrhagic cystitis

Small/Large intestine	None	Mild diarrhea; mild cramping; bowel movement 5 times daily; slight rectal discharge or bleeding	Moderate diarrhea and colic; bowel movement > 5 times daily; excessive rectal mucus or intermittent bleeding	Obstruction or bleeding, requiring surgery	Necrosis/Perforation Fistula

### Patient Follow-up

GU and lower GI symptoms and toxicity were prospectively assessed and graded by the physician at baseline and weekly during RT. Follow-up visits were at 4 weeks post-RT, and then every 4 months during the first year, every 6 months during the second and third years, and yearly thereafter. Each visit consisted of a medical history, physical examination including digital rectal exam, and serum PSA measurement. Quality of life and sexual function were assessed using the Expanded Prostate Cancer Index Composite (EPIC) questionnaire. These are not analyzed in this report.

### Sample Size and Statistical Analysis

Using the baseline assumption of 8.5% ≥ grade 2 late rectal toxicity, a sample size of 74 patients was required to show with a 95% confidence interval (95% CI) that the rate was equal or inferior to 15%. Target accrual was set at 78 to account for a 5% loss of patients at follow-up. All reported 95% confidence intervals are exact binomial. Overall survival, bDFS and cumulative toxicity rates were calculated by the actuarial method of Kaplan-Meier.

## Results

### Patient Characteristics and Treatment Delivery

Between March 2006 and August 2008, 81 patients were accrued in two institutions (Centre hospitalier de l'Université de Montréal and Centre hospitalier universitaire de Québec). One patient withdrew consent and opted for treatment with low dose rate brachytherapy. Patient characteristics are shown in Table 3. No patient received neoadjuvant, concurrent nor adjuvant hormone therapy.

**Table 3:** Patient baseline characteristics and delivered treatment

Median age (range)	70 years (56-77)
Clinical Stage	
T1b	1 (1%)
T1c	57 (71%)
T2a	22 (28%)
Gleason Score	
5	2 (2.5%)
6	79 (97.5%)
Median Initial PSA (range)	5.9 ng/ml (0.81-9.89)
Median prostate volume (range)*	41 cc (10-90)
Median Dose	45 Gy
Median Treatment Time	57 days

* as determined by TRUS or planning CT scan

As of July 2010, 80 patients had completed treatment with a minimum follow-up of 20 months. Median follow-up was 33 months (range, 20-51). All patients received the planned dose of 45 Gy in nine weekly fractions. There were no treatment interruptions. PTV coverage criteria were met for all plans. Dose constraints for the bladder were violated in four plans (55-68% of bladder received above 40 Gy). Minor protocol deviations in rectal dose constraints were found in three cases, consisting of the deviation of a single dose-to-percent volume constraint out of the three shown in Table 1. Dose constraints to femoral heads were met in all plans. In our cohort of patients, prostatic volume did not seem to be a factor in dosimetric violations for bladder or rectum. All but one patient with dosimetric violations had prostate volumes inferior to 50 cc.

### Toxicity

Acute GU toxicity during treatment was common with grade 0 in 38%, grade 1 in 29%, grade 2 in 29%, and grade 3 in 4%. No grade 4 acute GU toxicity occurred. Acute GI toxicity was grade 0 in 62%, grade 1 in 27%, grade 2 in 11%, and no grade 3 or 4 occurred during treatment. One month after treatment, most GU and GI symptoms had already regressed (Table 4). Crude toxicity rates on treatment, at 1 month and maximal acute toxicity are shown in Table 4.

**Table 4:** Acute urinary and rectal toxicity after prostate EBRT

RTOG GRADE					
	**0**	**I**	**II**	**III**	**IV**

GENITOURINARY					
	
On-treatment	30 (38%)	23 (29%)	23 (29%)	3 (4%)	-
	
1 month	54 (68%)	20 (25%)	5 (6%)	1 (1%)	-
	
Maximal	28 (35%)	23 (29%)	25 (31%)	4 (5%)	-

GASTROINTESTINAL					
	
On-treatment	49 (62%)	21 (27%)	9 (11%)	-	-
	
1 month	65 (81%)	13 (16%)	2 (3%)	-	-
	
Maximal	44 (56%)	24 (30%)	11 (14%)	-	-

Data for all and 52 patients were available for analysis at 19 and 31 months, respectively, and are shown in Table 5. Crude late grade ≥ 2 GI toxicity was 10% (95% CI, 5 to 19) and 8% (95% CI, 2 to 19) at 19 and 31 months, respectively. Cumulative late grade ≥ 3 GI toxicity at 3 years is 11%. All cases of late grade 3 GI toxicity consisted of rectal bleeding requiring endoscopic intervention (argon plasma coagulation), after which most symptoms resolved and patients returned to Grade 0 or 1 toxicity. No patient underwent surgery for rectal bleeding. Late grade 2 GU toxicity consisted mainly of moderate frequency and dysuria. There were no cases of late grade 3 GU toxicity. The only severe (grade 4) late GU toxicity was a case of severe hemorrhagic cystitis, requiring blood transfusions, four cystoscopies, discontinuation of blood thinners (aspirin) and eventually radical cysto-prostatectomy for bladder necrosis.

**Table 5:** Late urinary and rectal toxicity after prostate EBRT

RTOG GRADE					
	**0**	**I**	**II**	**III**	**IV**

GENITOURINARY					
	
19 months (n = 79)	74 (94%)	1 (1%)	3 (4%)	-	1 (1%)
	
31 months (n = 53)	47 (89%)	-	5 (9%)	-	1 (2%)
	
37 months (n = 28)	23 (82%)	1 (4%)	3 (11%)	-	1 (4%)

GASTROINTESTINAL					
	
19 months (n = 78)	61 (78%)	9 (12%)	4 (5%)	4 (5%)	-
	
31 months (n = 52)	36 (69%)	12 (23%)	3 (6%)	1 (2%)	-
	
37 months (n = 27)	20 (74%)	6 (22%)	1 (4%)	-	-

### Biochemical Response and Outcomes

**Figure 1:**
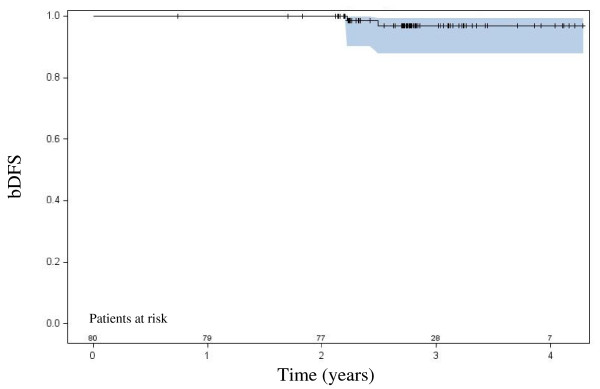
**Biochemical disease-free survival with 95% confidence interval**.

At last follow-up, 2 patients had a biochemical failure by the Phoenix definition, one with documented pelvic nodal relapse and the other has negative digital rectal exam, pelvic CT scan and bone scan. Both failures were documented at 31 months follow-up. The three-year actuarial biochemical control rate is 97%, as shown in Figure 1.

**Figure 2:**
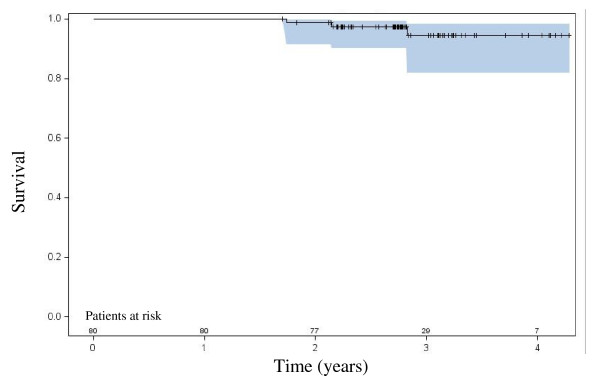
**Overall survival with 95% confidence interval**.

There have been no prostate cancer-related deaths at time of manuscript preparation. Three patients died of metastatic lung cancer of which none had a biochemical failure at last follow-up. The three-year actuarial overall survival rate is 94%, as shown in Figure 2.

## Discussion

Although most published data from phase I/II trial do not have sufficient follow-up to draw conclusions regarding the efficacy of hypofractionated regimens, early biochemical control is encouraging [11-13]. A recent phase III randomized trial [14] comparing hypofractionated (62 Gy/20 fractions/5 weeks) and conventional fractionation (80 Gy/40 fractions/8 weeks) in high-risk patients demonstrated equivalent late toxicity and superior 3-year freedom from biochemical failure (87% vs 79%, respectively) for the hypofractionated arm. Many factors in this trial may confound interpretation of the hypofractionation efficacy, such as the inclusion of high risk patients, and concomitant use of hormonal therapy for 9-months which may have had long-term castration effect in some patients. While this effect should in theory be balanced in the two arms, it does reduce the power to detect a difference considering the short median follow up of 32 months. Furthermore, cross-trial comparisons of differing fractionation schemes are limited. Hypofractionation has been the focus of two other large randomized controlled studies [15-17]. However, the biological doses used in both control and hypofractionated arms are inferior to current doses, rendering comparison of results difficult. At least three ongoing randomized trials are studying the effectiveness and toxicity of various hypofractionated RT regimens compared with standard RT fractionation.

Our findings show that a hypofractionated RT regimen consisting of 45 Gy in nine weekly fractions of five Gy each is both feasible and well tolerated. Several characteristics render the treatment regimen unique and are worth mentioning: the fractionation scheme and total treatment time employed, the total dose is one of the highest BED delivered in a hypofractionated external beam regimen and patients were treated without hormonal therapy. Also, neither IMRT nor stereotactic body RT were permitted in this study, thus allowing only the radiobiological basis for hypofractionation to be tested, and the toxicity outcomes were not influenced by these newer radiation treatment delivery techniques.

The primary endpoint of this study, late grade 2 or more GI toxicity, was 8% (crude) at 31 months and the cumulative rate at 3 years was 19%. Since median follow-up is 33 months, additional toxicity is not excluded, emphasising the importance of continued follow-up.

We found acute grade ≥ 3 GI and GU toxicity rates of 0% and 5%, respectively. Cumulative late grade ≥ 3 GI toxicity and GU toxicity were 11% and 1%. This compares to results of other published studies of hypofractionated EBRT using fraction sizes ≥ 3 Gy, with acute grade ≥ 3 GI and GU toxicity rates ranging from 0-5% [13,18-23] and late grade ≥ 3 toxicity ranging from 0-8% [11,13,19,20,23,24]. Table 6 compares late grade ≥ 2 rectal and urinary toxicity of prospective hypofractionated trials using large fraction sizes.

**Table 6:** Late rectal and urinary toxicity of prospective hypofractionation studies using ≥ 3 Gy per fraction

Author	Fractionation Schedule	Fraction Size (Gy)	EQD2 if α/β 1.5^†^	EQD2 if α/β 3^‡^	Grade ≥ 2 rectal toxicity	Grade ≥ 2 urinary toxicity
Present study	45 Gy/9	5	83.6	72.0	8%/19%^§^	11%/24%^§^

Martin *et al *(13)	60 Gy/20	3	77.1	72.0	6% (5 years)	10%

Rene *et al *(24)	66 Gy/22	3	84.9	79.2	25%*	32%*

Arcangeli *et al *(14)	62 Gy/20	3.1	81.5	75.6	17% (3 years)	14%

Coote *et al *(18)	57-60 Gy/19-20	3	73.3-77.1	68.4-72.0	9.5% (2 years)	8%

Madsen *et al *(20)	33.5 Gy/5	6.7	78.5	65.0	7.5%	20%

King *et al *(11)	36.25 Gy/5	7.25	90.6	74.3	15%	29%

^† ^EQD2 if α/β 1.5 = biologically effective dose in 2 Gy fractions assuming α/β ratio of 1.5

^‡^EQD2 if α/β 3 = biologically effective dose in 2 Gy fractions assuming α/β ratio of 3

^§^Crude rate at 31 months/Cumulative three-year rate

*Worse crude rate at anytime during follow-up

In order to compare the toxicity of this treatment with standard fractionation RT, one can estimate that the dose of 45 Gy/9 weekly fractions is radiobiologically equivalent to 83.6 Gy delivered in daily 2 Gy fractions, assuming an α/β ratio of 1.5 for prostate adenocarcinoma. For late effects on normal tissue, this corresponds to 72 Gy/36 fractions assuming an α/β ratio of 3. Thus, an increase in late normal tissue complications is not anticipated. Comparing with standard fractionation dose escalation trials, the Dutch trial to 78 Gy showed a 3-year cumulative incidence of late grade ≥ 3 GI and GU toxicity of 4.7% and 7%, respectively [25]. Similarly, long-term follow-up of the MDACC dose escalation trial to 78 Gy showed grade 3 bowel toxicity of 7%, and grade 3 GU toxicity of 4% [26].

Grade 4 toxicity is a known but infrequent complication of prostate radiation therapy [26]. Bladder and rectal DVHs were per protocol for the patient who developed severe cystitis. Of note, this same patient also developed grade 3 rectal toxicity. To our knowledge, late grade 4 toxicity has not been described with hypofractionation thus far. In dose escalation trials with standard 1.8-2 Gy fractions, late urinary and rectal toxicities seem to achieve a plateau at approximately 5 years post-treatment [26], while others have reported that late toxicity continues to develop between 5 and 10 years after completion of therapy [27,28]. Although at 19 months, ninety-four percent of our patients are free of late GU toxicity (grade 0) and no cases of grade 3 toxicity are observed, this complication is worrisome. This may be a reflection of an inherent radiosensitivity particular to this patient, such as a pathogenic ATM gene mutation, or may even represent erroneous alpha/beta ratio estimates, although this is less likely considering the mounting body of evidence supporting a low alpha/beta ratio for prostate adenocarcinoma.

There is data to support that the α/β ratio of the rectum is higher than the generic value of 3. Studies of RT in endometrial, cervical and prostate cancer [5,28-34] estimate it to be between 4 and 6, possibly as a result of consequential late effects. Thus, for late rectal injury, there is a dependency on total treatment time, and a relative independency on dose per fraction. In order to minimize acute rectal injury and to avoid consequential late effects, it is important to maintain a sufficient treatment time. For this reason, Fowler suggested that total treatment time not be less than five weeks [5], which guided the choice of schedule for this protocol.

This hypothesis is supported by data from the Stanford team who delivered 36.25 Gy in five fractions of 7.25 Gy using stereotactic body RT for localized prostate cancer. In this study, the first 21 patients were treated on 5 consecutive days, but the treatment schedule was subsequently modified to three fractions per week due to the rate of rectal toxicity. Increasing the time between fractions resulted in a significant reduction of severe late rectal toxicity [11]. Longer treatment time, as used in our protocol, should not have an adverse effect on tumor control since the unusually low α/β ratio for prostate adenocarcinoma makes these cells relatively independent of total treatment time. This is supported by analysis of the effect of overall treatment time on outcome in the RTOG 75-06 and 77-06 trials [35].

One limitation of our trial may be that patients were not treated using IMRT despite the now well documented reduction in GU and GI toxicity seen with this RT technique [28,36,37]. We chose not to allow the use of IMRT in our protocol as this treatment technique was not widely available in Canada at the time the study was initiated. Since neither IMRT nor stereotactic body RT were allowed in this study, the acceptable toxicity rates do not seem to result from improved radiation delivery techniques and support the radiobiological basis for hypofractionation. One can assume that the therapeutic ratio of our hypofractionated regimen will likely be enhanced with the routine use of IMRT but that needs to be addressed in a randomized controlled trial.

## Conclusions

A hypofractionated RT regimen consisting of 45 Gy in nine once weekly fractions is both feasible and well tolerated, but long term follow-up is necessary to fully assess late toxicity, tumour control and survival. In addition to the radiobiological advantages of this treatment on tumour control, hypofractionation offers important logistical and financial benefits, for both the patient and the health care system. At least three large prospective randomized trials of hypofractionation are currently underway and results are eagerly awaited. In the meantime, hypofractionated RT for the curative treatment of prostate cancer remains investigational.

## Competing interests

The authors declare that they have no competing interests.

## Authors' contributions

CM and CL conceived the study and participated in its design and coordination. They also acquired, analysed and interpreted data, and drafted the manuscript.

NB conceived the study and participated in its design.

EV and JPB participated in the study design and coordination, and acquired, analysed and interpreted data.

DN, HV, TVN, MCB and MJ acquired, analysed and interpreted data.

BF participated in the design of the study and performed the statistical analysis.

All authors read and approved the final manuscript.
